# Predator Becomes Prey: Records of Intraguild Predation Between Velvet Worms (Onychophora) and Arthropods (Arthropoda) With an Expanded Review of Predation on Velvet Worms

**DOI:** 10.1002/ece3.74614

**Published:** 2026-11-04

**Authors:** Osvaldo Villarreal, Leonardo Delgado‐Santa, Luis F. García

**Affiliations:** ^1^ Centro de Ecología Instituto Venezolano de Investigaciones Científicas (IVIC) Altos de Pipe Edo. Miranda Venezuela; ^2^ Instituto y Museo del Instituto de Zoología Agrícola, Facultad de Agronomía Universidad Central de Venezuela Maracay Venezuela; ^3^ Grupo de Investigación Ecdysis, Programa de Biología Universidad del Quindío Armenia Colombia; ^4^ Polo Agroforestal, CENUR Noreste Universidad de la República Ciudad Rivera Uruguay

**Keywords:** food‐web structure, natural history records, omnivorous predators, Opiliones, Orthoptera, predator–prey dynamics, trophic interactions

## Abstract

From 3954 citizen‐science observations screened, we report novel cases of predation by omnivorous arthropods on velvet worms, including the first confirmed records for harvestmen and a cave wētā. Using photographic evidence derived from citizen‐science observations, we document multiple predation events involving harvestmen (Opiliones: Cranaidae and Triaenonychidae) and a cave wētā (Orthoptera: Rhaphidophoridae), encompassing both intraguild predation and higher‐order opportunistic predation within cryptic terrestrial habitats. These interactions represent previously undocumented trophic links between phylogenetically distant lineages and expand the known repertoire of omnivorous arthropods as predators of onychophorans. Our findings document a previously unrecorded vulnerability in Onychophora, a group traditionally regarded as specialized predators defended by an adhesive slime system, and underscore the trophic flexibility of arthropod predators operating within leaf‐litter and subterranean ecosystems. Collectively, these observations document previously unrecorded trophic links in cryptic environments and illustrate the value of natural history data and citizen‐science platforms for detecting rarely documented interactions.

## Graphical abstract

This study reports the first confirmed cases of velvet worm (Onychophora) predation by omnivorous arthropods, specifically two harvestmen species (Opiliones: Cranaidae and Triaenonychidae) and a cave wētā (Orthoptera: Rhaphidophoridae), discovered through citizen‐science data. By contextualizing these records with an expanded literature review of historical onychophoran predators, we document previously unrecognized trophic links within leaf‐litter and subterranean ecosystems. These findings highlight the unexpected vulnerability of velvet worms to generalist consumers and underscore the significant trophic flexibility of opportunistic arthropod predators operating in cryptic environments.

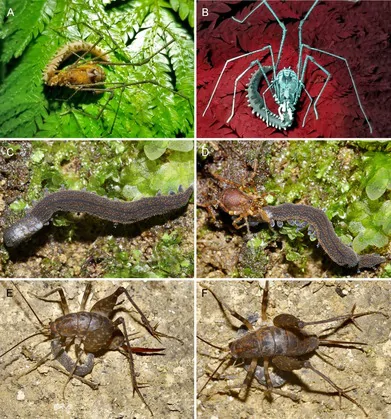

## 1 Introduction

Trophic interactions are fundamental components of ecological systems, as they structure biological communities and define the ecological roles of organisms within food webs (Brose et al. 2008; Thompson et al. 2012). A detailed understanding of these interactions is essential for identifying conservation priorities, particularly for species that exert a disproportionate influence on ecosystem functioning. Despite their importance, trophic interactions are often difficult to document in natural systems and have been approached using a variety of methodological strategies. Direct field observation is among the most traditional approaches and has been widely applied, especially in vertebrate ecology (Litvaitis 2000; Marion et al. 2020). More recently, molecular techniques such as PCR‐based gut content analyses and DNA metabarcoding have gained prominence, particularly for arthropods, where feeding events are rarely observed directly (Pompanon et al. 2012; Deagle et al. 2019). Nevertheless, direct observation remains a valuable complementary tool, as it is cost‐effective and capable of documenting rare, opportunistic, or unexpected trophic interactions that may be underrepresented in molecular datasets.

Arthropods play a central role as predators in many ecosystems and may act as top predators in certain terrestrial food webs, particularly at small spatial scales (Wise 1993; Polis et al. 2004). Although several arthropod groups, such as mantids and spiders, are considered obligate predators, many taxa traditionally regarded as omnivorous may also act as facultative predators. Insects such as crickets and cockroaches exhibit high protein requirements and frequently incorporate animal material into their diets through predation or scavenging (Coll and Guershon 2002; Walker and Masaki 1989; Toft et al. 2021). Similar trophic flexibility has been documented in arachnid groups, notably harvestmen (Opiliones), which consume a wide variety of food items including small invertebrates, fruits, fungi, and carrion (Acosta and Machado 2007; Powell et al. 2021). Consequently, arthropod groups often categorized as herbivorous or omnivorous may function as important scavengers and predators, underscoring the relevance of understanding their feeding habits for accurately characterizing terrestrial trophic webs.

Onychophora (velvet worms) represent a lineage of panarthropods and constitute tentatively the sister group to Arthropoda (Giribet and Edgecombe 2020). These rare and cryptic organisms inhabit soil and humid microhabitats and remain poorly studied from an ecological perspective (McCravy and Oliveira 2019), despite possessing a highly specialized and energetically expensive hunting apparatus (Morera‐Brenes and Monge‐Nájera 2010; Mayer et al. 2015; Oliveira and Mayer 2022). The adhesive slime may constitute up to 11% of a peripatus's body mass and requires approximately 24 days to replenish after depletion (Read and Hughes 1987). Velvet worms are traditionally regarded as obligate predators, relying on a distinctive adhesive slime system deployed as crossed jets to entrap small invertebrates (Mayer 2013; Mayer et al. 2015; Baer and Mayer 2012). Interactions between onychophorans and arachnids have been documented, including cases in which velvet worms feed on large spiders such as theraphosids or ctenids. In at least one instance (González et al. 2018), the ctenid spider appeared to be alive, suggesting active predation rather than scavenging. However, for other observations it remains unclear whether the prey was captured alive or scavenged. Anecdotal reports also include harvestmen being consumed by velvet worms (Monge‐Nájera et al. 1993; Dias and Lo‐Man‐Hung 2009). In addition, experimental studies have documented predation on spiders (e.g., ctenids) as part of their regular feeding behavior, noting that spiders are particularly alert prey that may occasionally counterattack and inflict damage (Read and Hughes 1987).

Despite their effectiveness as predators, records of natural enemies of velvet worms are remarkably scarce. Confirmed cases are largely restricted to a limited number of vertebrates, including birds (e.g., the Clay‐colored Thrush *Turdus grayi* ) and snakes (e.g., the coral snake *Micrurus hemprichii* , which preys almost exclusively on onychophorans, and *Trimetopon slevini* ), while spiders (e.g., *Ctenus* spp.) represent one of the few invertebrate groups documented as antagonistic interactors, often ambushing them before they can deploy their defensive slime (Monge‐Nájera et al. 1993; Franco and Monge‐Nájera 2016; Smokoska and Acosta‐Chaves 2017). The high cost of producing the defensive slime, coupled with their soft, unarmored bodies and slow locomotion, suggests that onychophorans may be particularly vulnerable to larger or opportunistic predators after exhausting their glue reserves, or when attacked by surprise (Read and Hughes 1987; Franco and Monge‐Nájera 2016). Indeed, the range of potential interactions may be broader than currently known, as scavenging arthropods such as harvestmen and ants have been observed feeding on the remains of prey items abandoned by feeding onychophorans (Read and Hughes 1987).

Although the adhesive slime of velvet worms constitutes a powerful defensive mechanism (Morera‐Brenes and Monge‐Nájera 2010), the diversity and ecological relevance of their natural enemies remain poorly understood. Given their soft, unarmored bodies and relatively slow locomotion, onychophorans may be vulnerable to larger opportunistic predators, similarly to other vermiform arthropods (Hopkin and Read 1992; Lewis 1981).

Here, we document the first confirmed cases of predation by omnivorous arthropods on velvet worms, adding previously unrecognized trophic interactions within leaf‐litter communities.

## 2 Material and Methods

### 2.1 Data Collection and Screening

A systematic search for potential predatory interactions (including intraguild predation, facultative predation, and scavenging) was conducted using the citizen‐science platform iNaturalist (www.inaturalist.org). On 24 December 2025, the taxon filter “Onychophora” was applied, yielding 3954 research‐grade photographic observations. Research Grade is an iNaturalist designation indicating that an observation has met the platform's community‐based identification and data‐completeness criteria. It was used here only as an initial database filter and not as independent verification of species identity or of the interaction. These records were manually reviewed to identify images depicting interspecific predatory interactions involving velvet worms.

### 2.2 Systematic Literature Search

We searched web‐indexed scholarly sources, publisher platforms, and institutional repositories from inception through 6 April 2026. The primary Boolean query combined (“Onychophora” OR onychophoran* OR “velvet worm*” OR *Peripatus* OR Peripatidae OR Peripatopsidae) with (predat* OR prey OR feeding OR diet OR ingest* OR consum* OR “stomach contents” OR “gut contents”). Equivalent searches were run in Spanish and Portuguese (onicóforo*, gusano de terciopelo, predación, depredación, presa, alimentación, predação, and conteúdo estomacal), together with predator‐specific terms (*Ctenus*, *Micrurus*, *Trimetopon*, *Urotheca*, *Turdus*, *Gallirallus*, *Porphyrio*, *Craugastor*, Opiliones, harvestman, centipede, chilopod, Orthoptera, and wētā/weta). We screened titles, abstracts, full texts, bibliographies, and citation trails from the core onychophoran natural‐history papers. We included traceable published or publicly archived records consisting of a direct observation or photograph, a stomach/gizzard or gut‐content record, or an explicit field report of an organism consuming a live or recently killed onychophoran. We excluded reverse interactions in which onychophorans consumed other animals, general statements without a traceable record, unverified citizen‐science observations, and records that documented only scavenging without evidence that the onychophoran had been killed or consumed as prey. Fourteen publications or publicly archived sources met these criteria as primary or directly traceable records.

### 2.3 Taxonomic Identification

Taxonomic identifications for all organisms follow the determinations provided by multiple iNaturalist users, whose collective expertise contributed to the research‐grade status of the observations. For the Neotropical onychophoran, only three genera have been recorded from Ecuador: *Oroperipatus* Cockerell, 1908, *Peripatus* Guilding, 1826 *and Epiperipatus* Clark, 1913 (Luzuriaga‐Rodríguez et al. 2025). Given the difficulties of identifying onychophorans from photographs alone, the iNaturalist community's suggestion was followed as a tentative identification as *Oroperipatus* sp., while acknowledging that two other genera are known from the country. According to Luzuriaga‐Rodríguez et al. (2025), no previous record of Onychophora exists for Manabí province (the nearest documented occurrences are from Guayas province, e.g., *O. quitensis* ); therefore, our observation from Jama‐Coaque represents the first onychophoran record for Manabí, albeit identified only tentatively to genus level due to the lack of voucher specimens. For the New Zealand records, the species *Peripatoides novaezealandiae* (Hutton, 1876) is as indicated in the iNaturalist database. Harvestmen were identified to genus level by one of the authors (O.V.) for *Ventrivomer* sp. (Cranaidae) based on diagnostic morphological characters visible in high‐reolution images, while *Nuncia* (*Nuncia*) *conjuncta* (Triaenonychidae) retains its iNaturalist community identification. We acknowledge that low taxonomic resolution is a limitation of photographic and citizen‐science data. In this study, however, the ecological interpretation does not depend on species‐level identification: all onychophorans are predatory members of the same trophic guild, so identification to Onychophora is sufficient to document the interaction. Accordingly, the resolution strictly required here is “Onychophora” for the prey and the respective arthropod order for the predators; generic and specific names are retained for contextual identifications only.

### 2.4 Estimation of Prey: Predator Size Relationships

Prey:predator size relationships were estimated using relative body proportions observed within the same photographic frame. Measurements were based on maximum visible body length and expressed as approximate size ratios. Because no scale reference was available in the images, these estimates were made visually by the authors and should be interpreted as approximate comparative estimates rather than precise morphometric values.

## 3 Results

Of the 3954 citizen‐science observations screened, three independent arthropod consumption events involving velvet worms were identified and analyzed. The predators comprised two species of harvestmen (Cranaidae and Triaenonychidae) and a cave wētā (Orthoptera: Rhaphidophoridae). A summary of these interactions is reported in Table 1, including the prey:predator size ratio for each event. This denominator is not a probability sample and may overrepresent unusual or photographable events.

**TABLE 1:** Arthropod predators of different velvet worm species. (*Size ratio not measurable due to photo angle.) Prey:Predator size ratio: Values > 1 indicate that the prey is larger than the predator; values < 1 indicate that the prey is smaller.

Onychophoran species	Predator species	Predator order	Source	Location	Prey:Predator size ratio
*Epiperipatus* sp.	*Ctenus* spp.	Araneae	Franco and Monge‐Nájera (2016)	Colombia	3.20
*Epiperipatus* sp.	Ctenidae	Araneae	Franco and Monge‐Nájera (2016)	Costa Rica	*
*Peripatoides novaezealandiae*	*Nuncia conjuncta*	Opiliones	iNaturalist	New Zealand	~4.1
*Oroperipatus* sp.	*Ventrivomer* sp.	Opiliones	iNaturalist	Ecuador	1.90
*Peripatoides novaezealandiae*	Cave wētā (*Miotopus diversus*)	Orthoptera	iNaturalist	New Zealand	0.44

### 3.1 Opiliones

The first event was documented on 24 April 2022 in the Jama‐Coaque Reserve, Pedernales, Manabí Province, Ecuador (−0.1229°, −80.1227°; alt. 554 m). An adult female harvestman (Figure 1A,B) *Ventrivomer* sp. (Opiliones: Cranaidae) was observed consuming an *Oroperipatus* sp. (Onychophora: Peripatidae) velvet worm on a mat of *Selaginella* sp. The harvestman grasped the onychophoran with its pedipalps and applied the chelicerae to the prey's body. The onychophoran appeared intact, and no adhesive slime was visible on the predator or surrounding substrate.

**FIGURE 1:**
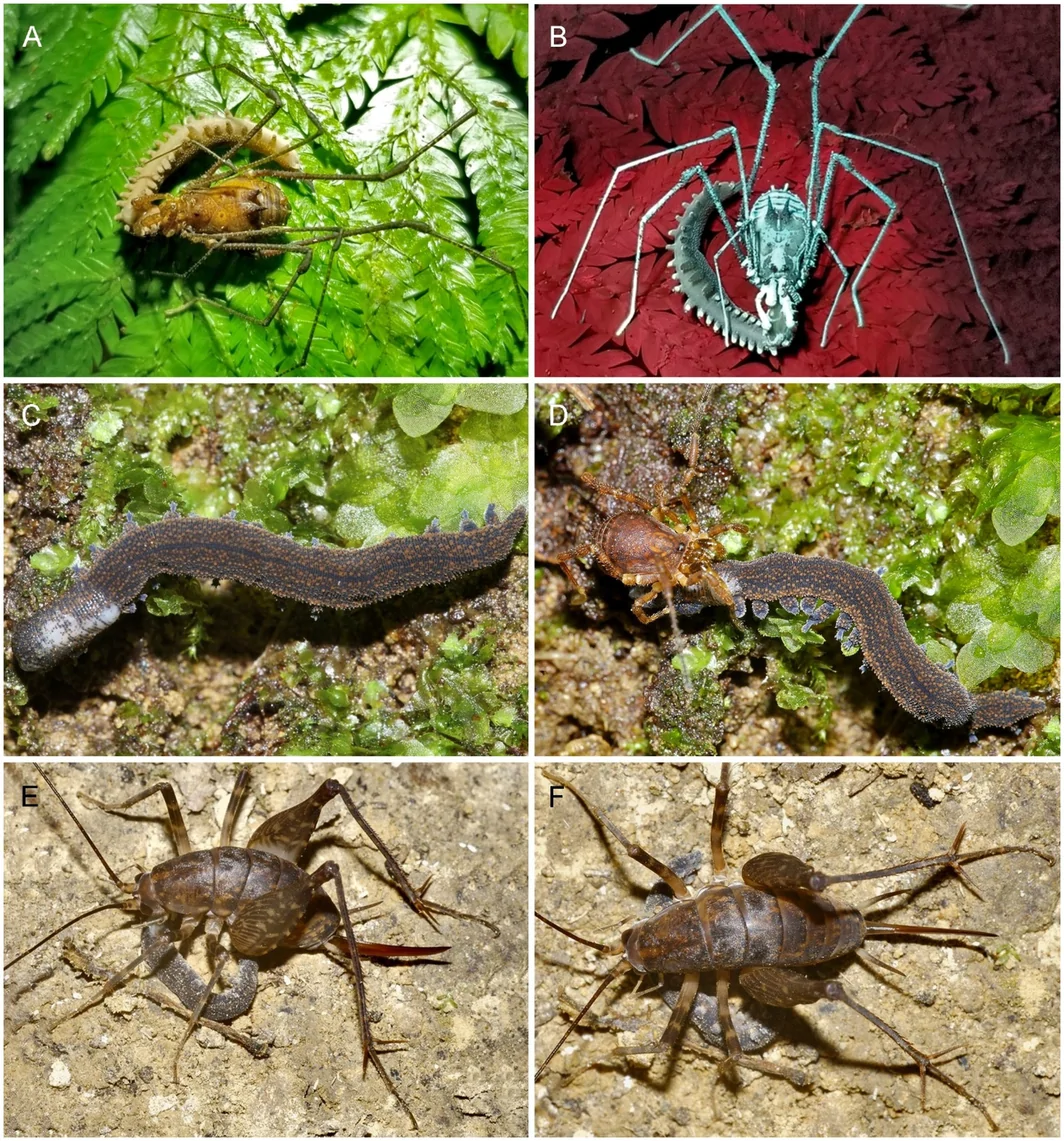
Feeding events involving velvet worms (Onychophora) and omnivorous arthropods. (A, B) Ventrivomer sp. (Opiliones: Cranaidae) consuming Oroperipatus sp. in Ecuador. (C, D) Nuncia (Nuncia) conjuncta (Opiliones: Triaenonychidae) consuming *Peripatoides novaezealandiae* in New Zealand. (E, F) Cave wētā Miotopus diversus (Orthoptera: Rhaphidophoridae) consuming *Peripatoides novaezealandiae* in New Zealand. (A, B) Mike Ellis, via iNaturalist observation 112,910,966. (C, D) and (E, F) Uwe Schneehagen, via iNaturalist observations 88,363,312 and 105,486,552, respectively. Photographs reproduced with permission and/or under the applicable Creative Commons licenses.

A second event involved a harvestman (Figure 1C,D) *Nuncia (Nuncia) conjuncta* Forster, 1954 (Opiliones: Triaenonychidae), recorded on 24 July 2021 near Lower Hutt, Wellington (−41.1830°, 174.9125°, alt. 171 m), New Zealand. Two images taken approximately 20 min apart show the harvestman feeding on a velvet worm ( *Peripatoides novaezealandiae* ) on a moss‐covered rock substrate. The velvet worm was already severely damaged when first observed, its body partially torn and the harvestman's mouthparts engaged near the anterior region. The prey:predator size ratio was approximately 4–5:1 (the velvet worm was four to five times the body length of the harvestman). Whether the harvestman encountered a moribund individual or actively captured it alive cannot be determined from the images. The public discussion associated with this observation on iNaturalist raised scavenging as a possible interpretation.

### 3.2 Orthoptera

A third event documents a female cave wētā (Figure 1E,F), *Miotopus diversus* (Orthoptera: Rhaphidophoridae), recorded on 22 January 2022 near Wilton, Wellington (−41.2668°, 174.7560°, alt. 78 m), New Zealand preying upon *Peripatoides novaezealandiae* (Onychophora: Peripatopsidae). The photograph depicts the wētā actively consuming an intact, fully extended velvet worm, holding it with the forelegs while feeding on the anterior region. The prey:predator size ratio was approximately 0.44 (the onychophoran was smaller than the wētā).

### 3.3 Systematic Literature Search

After screening and deduplication, 15 traceable candidate documents were examined in the evidence matrix (Table 2). Fourteen met the inclusion criteria as primary or directly traceable evidence and were retained for synthesis. One additional source, the New Zealand Department of Conservation (2014), was retained only to trace a secondary report of predation by the North Island robin ( *Petroica australis longipes* ) and was not counted as an independent record.

**TABLE 2:** Traceable published and publicly archived records of organisms reported to prey on onychophorans before this study. Records from the present study are presented in Table 1.

No.	Source	Predator	Prey and locality	Reported evidence	Evidence type
1	Carroll (1963)	*Gallirallus australis*	*Peripatus*; New Zealand	Stomach contents from 86 wekas; the peripatus record is repeated by DOC 2014.	Primary/stomach contents
2	Carroll (1966)	*Porphyrio melanotus*	*Peripatus novaezealandiae* ; New Zealand	298 gizzards examined; peripatus found occasionally.	Primary/gizzards
3	Greene (1973)	*Micrurus hemprichii/ortoni*	Onychophorans; Neotropics	Thesis on the diet and feeding behavior of coral snakes.	Primary/diet
4	Dixon and Soini (1977)	*Micrurus hemprichii ortoni*	*Peripatus*; Loreto, Peru	Two Peripatus reported in one stomach.	Primary/stomach contents
5	Cunha and Nascimento (1978)	*Micrurus* sp.	Onychophorans; eastern Amazonia	Diet record cited by later syntheses.	Traceable/primary source
6	Dyrcz (1982)	*Turdus grayi*	Onychophorans; Panama	Adults observed carrying onychophorans to nestlings.	Primary/direct observation
7	Roze (1982)	*Micrurus* sp.	*Peripatus*; Neotropics	Taxonomic and biological synthesis of coral snakes.	Secondary/synthesis
8	Cunha and Nascimento (1982)	*Micrurus* sp.	Onychophorans; Pará and Maranhão, Brazil	Natural‐history record cited by later syntheses.	Traceable/primary source
9	Monge‐Nájera et al. (1993)	*Turdus grayi; Micrurus* sp.	Onychophorans; tropical America	Synthesis recovering field and diet records.	Secondary/traceable synthesis
10	Martins and Oliveira (1998)	*Micrurus hemprichii*	Onychophorans; Manaus, Brazil	Snake diet record from forest natural history	Primary/natural history
11	Franco and Monge‐Nájera (2016)	*Ctenus* spp.	*Epiperipatus*; Colombia and Costa Rica	Two photographs: one immobilized specimen and one dead specimen; the latter may represent scavenging.	Primary/photographs; mixed evidence
12	Smokoska and Acosta‐Chaves (2017)	*Trimetopon slevini*	Onychophoran; Costa Rica	Snake extracting and consuming a live onychophoran in a photographic observation.	Primary/direct predation
13	Méndez Fallas and Solórzano (2024)	*Urotheca guentheri*	*Epiperipatus* sp.; Costa Rica	Snake consuming a live onychophoran; photograph and coordinates provided.	Primary/direct predation
14	Cabezas Pérez et al. (2026)	*Craugastor fitzingeri*	*Macroperipatus valerioi* ; Costa Rica	First published observation of a frog consuming an onychophoran.	Primary/direct predation
15	New Zealand Department of Conservation (2014)	*Petroica australis longipes*	*Peripatus*; New Zealand	Secondary mention of predation attributed to Carroll 1963; not an independent primary record.	Secondary only/not independent

The 14 eligible sources documented eight predator taxa or taxonomic lineages: the Clay‐colored Thrush ( *Turdus grayi* ), North Island weka ( *Gallirallus australis* ), pukeko (*Porphyrio melanotus*), coral snakes in the *Micrurus hemprichii/ortoni* complex, *Ctenus* spp., *Trimetopon slevini* , *Urotheca guentheri* , and the Common Rainfrog ( *Craugastor fitzingeri* ). Evidence consisted of direct field observations or photographs for *Ctenus*, *Trimetopon*, *Urotheca*, and *Craugastor*; stomach, gizzard, or diet records for *Micrurus*, *Gallirallus*, and *Porphyrio*; and a direct field observation of *Turdus grayi* carrying onychophorans to nestlings. The review did not locate an eligible published record involving harvestmen or Rhaphidophoridae orthopterans, supporting the novelty of the observations reported here. Because several sources summarized diets or repeated earlier records, we report unique sources and predator taxa rather than potentially duplicated feeding events, and we did not perform a quantitative meta‐analysis.

## 4 Discussion

Interpreting these interactions from static imagery requires caution. Although the precise behavioral sequence—active predation versus opportunistic scavenging—cannot be definitively resolved, several lines of evidence suggest that the documented events likely represent predation. For cursorial predators such as harvestmen and cave wētā, the interpretation leans toward predation, though scavenging on moribund individuals cannot be ruled out. The intact, non‐decayed state of the onychophorans in multiple, independent observations argues against scavenging on long‐dead carcasses, a point reinforced by experimental data on onychophoran decay: internal organs begin to break down within hours and are completely lost by approximately 8–12 days post‐mortem, after which the body cavity contains only a disaggregated, soapy substance (Murdock et al. 2014). A carcass older than this would therefore be a very different resource from the intact individuals observed here. We cannot entirely rule out that the prey were moribund or that the interactions were influenced by human intervention—that is, that an organism or carcass could have been moved, placed, or otherwise presented in a way that altered the natural interaction before the photograph was taken. We have no evidence that this occurred; it is an epistemic limitation inherent in interpreting isolated photographs uploaded by third parties, and we do not imply any allegation against the observers. For the New Zealand harvestman event in particular, the associated iNaturalist discussion raised scavenging as a possibility; we acknowledge this and treat scavenging as an alternative interpretation, since the behavioral sequence cannot be reconstructed from static images. Despite this uncertainty, the convergence of this interaction across two phylogenetically distant harvestman families (Cranaidae and Triaenonychidae) and an orthopteran suggests a rarely documented, predatory capacity toward velvet worms within generalist arthropod diets. Ultimately, whether classified as strict predation or scavenging of moribund individuals, these records establish novel trophic links between velvet worms and higher‐level arthropod consumers.

Our observations provide novel evidence of natural enemies of velvet worms and contribute new insights into the trophic roles of omnivorous arthropods within cryptic terrestrial ecosystems. Records involving harvestmen expand the known trophic breadth of Opiliones, illustrate an unrecognized role of Onychophora as prey, and document intraguild nature of these interactions in leaf‐litter and subterranean food webs. Such intraguild interactions, where species can switch roles as predator and prey depending on context, are increasingly recognized as widespread in terrestrial ecosystems (Polis et al. 1989). For instance, while frogs are typical predators of many arthropods, harvestmen have recently been documented preying upon frogs in the Neotropics (e.g., Castanho and Pinto‐da‐Rocha 2005; Villarreal Manzanilla et al. 2008), and similarly, dragonfly larvae prey on tadpoles while adult frogs prey on metamorphosing dragonflies (Beranek et al. 2023). This fluidity challenges static trophic classifications and suggests that in cryptic habitats, the outcome of encounters between generalist predators may often depend simply on which organism gains the initial advantage (Polis 1981; Polis and McCormick 1987).

Large Neotropical harvestmen of the family Cranaidae exemplify marked trophic plasticity. Although historically characterized as omnivores or scavengers, they are now known to prey upon a wide range of organisms, including vertebrates such as frogs (Hayes 1983; Castanho and Pinto‐da‐Rocha 2005; Villarreal Manzanilla et al. 2008). Our record extends this pattern by documenting predation on another predatory lineage. Velvet worms are rarely documented as prey, which may reflect their generally low abundance in natural habitats, their cryptic habits, or their sophisticated chemical defense based on the rapid ejection of adhesive slime, which functions both in prey capture and deterrence of aggressors (Morera‐Brenes and Monge‐Nájera 2010; Mayer 2013). The ability of *Ventrivomer* sp. to overcome this defense is consistent with active intraguild predation, although scavenging cannot be excluded.

The occurrence of onychophoran predation in two distantly related harvestman lineages—*Ventrivomer* sp. (Cranaidae) in the Neotropics and *Nuncia conjuncta* (Triaenonychidae) in New Zealand—illustrates that this trophic interaction is not confined to a single family. This pattern is consistent with the flexible nutritional strategies of omnivorous predators, which may exploit rare or unconventional prey when opportunities arise (Toft et al. 2021). These observations cannot distinguish between ecological flexibility, parallel adaptation, or simple encounter context, and we do not infer any specific evolutionary scenario. Accordingly, these records suggest that onychophorans fall within the prey spectrum of large‐bodied Laniatores, adding to the evidence for flexible trophic roles in cryptic terrestrial food webs.

Predation by the cave wētā *Miotopus diversus* follows a similar ecological pattern. Crickets are increasingly recognized as omnivorous carnivores whose diets may include substantial amounts of animal prey, particularly under conditions of nutritional limitation (Walker and Masaki 1989; Ogita et al. 2021; Toft et al. 2021). Although *Peripatoides novaezealandiae* is itself a predator of small invertebrates, in this case it served as prey to a larger arthropod occupying overlapping cave and edaphic microhabitats.

This observation contrasts with and complements the only previously documented case of spider‐onychophoran predation, which involved a wandering spider (*Ctenus* spp., Ctenidae) preying on *Epiperipatus* in a Neotropical forest floor context (Franco and Monge‐Nájera 2016). Unlike many arthropod prey that possess a rigid exoskeleton often coupled with chemical defenses, onychophorans lack a hardened exoskeleton and rely primarily on their adhesive slime for defense (Read and Hughes 1987; Morera‐Brenes and Monge‐Nájera 2010; Mayer et al. 2015). Consequently, velvet worms may be comparatively more vulnerable to spider attacks. These traits likely reduce the mechanical and chemical challenges faced by spiders when subduing velvet worms.

Taken together, the focused literature search indicates that onychophorans may serve as occasional prey within a sparse and heterogeneous set of vertebrate and arthropod interactions. Although 14 of 15 traceable documents met our inclusion criteria, the available evidence is too limited and uneven to infer interaction frequency, population‐level importance, or whether sex‐specific ecological requirements or predator specialization play a role. The absence of previous eligible records for harvestmen and Rhaphidophoridae orthopterans therefore underscores the novelty of our observations, which should be regarded as previously undocumented trophic links rather than evidence of common predation. Within this context, sex‐specific activity, foraging mode, and life‐history timing may deserve attention in future records: mate‐searching males or gravid females may move differently, active hunters in the leaf litter may encounter onychophorans more often than ambush predators, and molting or reproduction may create brief windows of vulnerability. We mention these only as tentative possibilities. Sex identification in onychophorans is difficult from photographs, unlike taxa with clear sexual dimorphism, so even fewer inferences are possible from the present records.

Taken together, these observations support the view that arthropod predators occupy flexible trophic roles and that velvet worms, despite their specialized predatory adaptations, are embedded within complex intraguild interactions in cryptic leaf‐litter and subterranean ecosystems. The limited number of records may reflect observational bias, but the present data do not allow inference about frequency or food‐web effects. These observations nevertheless illustrate the value of detailed natural history records for documenting rarely observed trophic links.

## Author Contributions

**Osvaldo Villarreal:** conceptualization (lead), data curation (equal), investigation (equal), methodology (equal), resources (equal), supervision (equal), visualization (equal), writing – original draft (equal), writing – review and editing (equal). **Leonardo Delgado‐Santa:** conceptualization (supporting), data curation (equal), investigation (equal), methodology (equal), resources (equal), visualization (equal), writing – original draft (supporting), writing – review and editing (equal). **Luis F. García:** conceptualization (equal), data curation (equal), investigation (lead), methodology (equal), resources (equal), visualization (supporting), writing – original draft (lead), writing – review and editing (equal).

## Conflicts of Interest

The authors declare no conflicts of interest.

## Data Availability

The photographic records supporting this study are openly available on the iNaturalist platform. Licenses were checked individually for each observation: observation 112,910,966 (https://www.inaturalist.org/observations/112910966) is released under CC BY‐NC 4.0; observation 88,363,312 (https://www.inaturalist.org/observations/88363312), and observation 105,486,552 (https://www.inaturalist.org/observations/105486552) are released under CC BY‐SA 4.0. No other data were generated or analyzed during this study.

## References

[ece374614-bib-0001] Acosta, L. E. , and G. Machado . 2007. “Diet and Foraging.” In Harvestmen: The Biology of Opiliones, edited by R. Pinto‐da‐Rocha , G. Machado , and G. Giribet , 309–338. Harvard University Press.

[ece374614-bib-0002] Baer, A. , and G. Mayer . 2012. “Comparative Anatomy of Slime Glands in Onychophora (Velvet Worms).” Journal of Morphology 273, no. 10: 1079–1088. 10.1002/jmor.20044. 22707384

[ece374614-bib-0003] Beranek, C. T. , J. Clulow , and M. Mahony . 2023. “Life Stage Dependent Predator–Prey Reversal Between a Frog ( *Litoria aurea* ) and a Dragonfly (*Anax papuensis*).” Ecology 104, no. 8: e4115. 10.1002/ecy.4108. 37212489

[ece374614-bib-0004] Brose, U. , R. B. Ehnes , B. C. Rall , O. Vucic‐Pestic , E. L. Berlow , and S. Scheu . 2008. “Foraging Theory Predicts Predator–Prey Energy Fluxes.” Ecology 77: 1072–1078. 10.1111/j.1365-2656.2008.01408.x. 18540967

[ece374614-bib-0005] Cabezas Pérez, G. , E. Moya Jiménez , A. Muñoz Mora , and R. Nuñez Escalante . 2026. “First Documented Case of a Common Rainfrog ( *Craugastor fitzingeri* ) Preying on a Velvet Worm ( *Macroperipatus valerioi* ) in Uvita, Puntarenas, Costa Rica.” Reptiles & Amphibians 33: e23884. 10.17161/randa.v33i1.23884.

[ece374614-bib-0006] Carroll, A. L. K. 1963. “Food Habits of the North Island Weka.” Notornis 10, no. 6: 281–284, 289–300.

[ece374614-bib-0007] Carroll, A. L. K. 1966. “Food Habits of Pukeko (*Porphyrio melanotus* Temminck).” Notornis 13, no. 3: 133–141. 10.63172/017390bjrkob.

[ece374614-bib-0008] Castanho, L. M. , and R. Pinto‐da‐Rocha . 2005. “Harvestmen (Opiliones: Gonyleptidae) Predating on Treefrogs (Anura: Hylidae).” Revista Ibérica de Aracnología 11: 43–45.

[ece374614-bib-0009] Coll, M. , and M. Guershon . 2002. “Omnivory in Terrestrial Arthropods: Mixing Plant and Prey Diets.” Annual Review of Entomology 47: 267–297. 10.1146/annurev.ento.47.091201.145209. 11729076

[ece374614-bib-0010] Cunha, O. R. , and F. P. Nascimento . 1978. “Ofídios da Amazônia X: As cobras da região leste do Pará.” Publicações Avulsas do Museu Paraense Emílio Goeldi 31: 1–218.

[ece374614-bib-0011] Cunha, O. R. , and F. P. Nascimento . 1982. “Ofídios da Amazônia XIV: As espécies de *Micrurus*, *Bothrops*, *Lachesis* e *Crotalus* do sul do Pará e oeste do Maranhão, incluindo áreas de cerrado deste estado (Ophidia: Elapidae e Viperidae).” Boletim do Museu Paraense Emílio Goeldi, Série Zoologia 112: 1–58.

[ece374614-bib-0012] Deagle, B. E. , A. C. Thomas , J. C. McInnes , et al. 2019. “Counting With DNA in Metabarcoding Studies: How Should We Convert Sequence Reads to Dietary Data?” Molecular Ecology 28, no. 2: 391–406. 10.1111/mec.14734. PMC6905394 29858539

[ece374614-bib-0013] Dias, S. C. , and N. F. Lo‐Man‐Hung . 2009. “First Record of an Onychophoran (Onychophora, Peripatidae) Feeding on a Theraphosid Spider (Araneae, Theraphosidae).” Journal of Arachnology 37: 116–117. 10.1636/ST08-20.1.

[ece374614-bib-0014] Dixon, J. R. , and P. Soini . 1977. “The Reptiles of the Upper Amazon Basin, Iquitos Region, Peru. Part II: Crocodilians, Turtles, and Snakes.” Milwaukee Public Museum, Contributions in Biology and Geology 12: 1–91.

[ece374614-bib-0015] Dyrcz, A. 1982. “Synanthropy in Tropics as Studied in Clay‐Coloured Robin *Turdus grayi* on Panama Lowland.” Materials of the International Conference “Animals in the Urbanized World,” Jabłonna, October 1979. Ossolineum, 27–37.

[ece374614-bib-0016] Franco, R. , and J. Monge‐Nájera . 2016. “Inverted Roles: Spider Predation Upon Neotropical Velvet Worms.” Cuadernos de Investigación UNED 8: 171–173.

[ece374614-bib-0049] Giribet, G. , and G. D. Edgecombe . 2020. The Invertebrate Tree of Life. Princeton University Press. 10.2307/j.ctvscxrhm.

[ece374614-bib-0017] González, M. , Á. Sosa‐Bartuano , and J. Monge‐Nájera . 2018. “A Velvet Worm (Onychophora: Peripatidae) Feeding on a Free Ranging Spider in Sierra Llorona, Panama.” Cuadernos de Investigación UNED 10, no. 2: 283–284. 10.22458/urj.v10i2.2159.

[ece374614-bib-0018] Greene, H. W. 1973. “The Food Habits and Feeding Behavior of New World Coral Snakes.” M.Sc. thesis, University of Texas at Arlington.

[ece374614-bib-0019] Hayes, M. P. 1983. “Predation on the Adults and Prehatching Stages of Glass Frogs (Centrolenidae).” Biotropica 15, no. 1: 74–76. 10.2307/2388005.

[ece374614-bib-0020] Hopkin, S. P. , and H. J. Read . 1992. The Biology of Millipedes. Oxford University Press.

[ece374614-bib-0021] Lewis, J. G. E. 1981. The Biology of Centipedes. Cambridge University Press.

[ece374614-bib-0022] Litvaitis, J. A. 2000. “Investigating Food Habits of Terrestrial Vertebrates.” In Research Techniques in Animal Ecology: Controversies and Consequences, edited by L. Boitani and T. K. Fuller , 165–190. Columbia University Press.

[ece374614-bib-0050] Luzuriaga‐Rodríguez, N. , M. Calle‐Morán , H. Gaona‐Garrido , and J. Guanuche‐Zhicay . 2025. “Registro de siete especies de onicóforos en la provincia de Zamora Chinchipe, Amazonía sur ecuatoriana.” Acta Oceanográfica del Pacífico 7, no. 1: 40–59.

[ece374614-bib-0023] Marion, S. , A. Davies , U. Demšar , R. J. Irvine , P. A. Stephens , and J. Long . 2020. “A Systematic Review of Methods for Studying the Impacts of Outdoor Recreation on Terrestrial Wildlife.” Global Ecology and Conservation 22: e00917. 10.1016/j.gecco.2020.e00917.

[ece374614-bib-0024] Martins, M. , and M. E. Oliveira . 1998. “Natural History of Snakes in Forests of the Manaus Region, Central Amazonia, Brazil.” Herpetological Natural History 6: 78–150.

[ece374614-bib-0025] Mayer, G. 2013. “Structure and Development of Onychophoran Adhesive Glands.” Journal of Morphology 274: 1243–1261.

[ece374614-bib-0026] Mayer, G. , I. S. Oliveira , A. Baer , J. U. Hammel , J. Gallant , and R. Hochberg . 2015. “Capture of Prey, Feeding, and Functional Anatomy of the Jaws in Velvet Worms (Onychophora).” Integrative and Comparative Biology 55, no. 2: 217–227. 10.1093/icb/icv004. 25829018

[ece374614-bib-0027] McCravy, K. W. , and I. S. Oliveira . 2019. “Onychophorans (Velvet Worms): Natural History and Conservation of a Vulnerable Invertebrate Taxon in the Caribbean Islands.” Caribbean Naturalist Special Issue No. 2: 135–155.

[ece374614-bib-0028] Méndez Fallas, J. , and A. Solórzano . 2024. “Predation on a Velvet Worm, *Epiperipatus* sp. (Onychophora: Peripatidae), by a Striped Glasstail, *Urotheca guentheri* (Squamata: Dipsadidae), in Costa Rica.” Reptiles & Amphibians 31: e22380. 10.17161/randa.v31i1.22380.

[ece374614-bib-0029] Monge‐Nájera, J. , Z. Barrientos , and F. Aguilar . 1993. “Behavior of *Epiperipatus biolleyi* .” Revista de Biología Tropical 41: 689–696.

[ece374614-bib-0030] Morera‐Brenes, B. , and J. Monge‐Nájera . 2010. “A New Giant Species of Placented Worm and the Mechanism by Which Onychophorans Weave Their Nets (Onychophora: Peripatidae).” Revista de Biología Tropical 58, no. 4: 1127–1142. 10.15517/rbt.v58i4.5398. 21246983

[ece374614-bib-0031] Murdock, D. J. E. , S. E. Gabbott , G. Mayer , and M. A. Purnell . 2014. “Decay of Velvet Worms (Onychophora), and Bias in the Fossil Record of Lobopodians.” BMC Evolutionary Biology 14, no. 222: 1–9. 10.1186/s12862-014-0222-z. PMC4266977 25472836

[ece374614-bib-0032] New Zealand Department of Conservation . 2014. New Zealand Peripatus/Ngaokeoke: Current Knowledge, Conservation and Future Research Needs. Department of Conservation.

[ece374614-bib-0033] Ogita, S. , Y. Tanaka , and T. Kuriwada . 2021. “Effect of Diet on Body Size and Survival of Omnivorous Crickets.” Entomological Science 24, no. 4: 347–353. 10.1111/ens.12487.

[ece374614-bib-0034] Oliveira, I. S. , and G. Mayer . 2022. “Phylum Onychophora.” In Invertebrate Zoology: A Tree of Life Approach, edited by B. Schierwater and R. DeSalle , 459–475. CRC Press.

[ece374614-bib-0035] Polis, G. A. 1981. “The Evolution and Dynamics of Intraspecific Predation.” Annual Review of Ecology, Evolution, and Systematics 12: 225–251. 10.1146/annurev.es.12.110181.001301.

[ece374614-bib-0036] Polis, G. A. , and S. J. McCormick . 1987. “Intraguild Predation and Competition Among Desert Scorpions.” Ecology 68, no. 2: 332–343. 10.2307/1939264.

[ece374614-bib-0037] Polis, G. A. , C. A. Myers , and R. D. Holt . 1989. “The Ecology and Evolution of Intraguild Predation: Potential Competitors That Eat Each Other.” Annual Review of Ecology and Systematics 20: 297–330. 10.1146/annurev.es.20.110189.001501.

[ece374614-bib-0038] Polis, G. A. , M. E. Power , and G. R. Huxel , eds. 2004. Food Webs at the Landscape Level. University of Chicago Press.

[ece374614-bib-0039] Pompanon, F. , B. E. Deagle , W. O. C. Symondson , D. S. Brown , S. N. Jarman , and P. Taberlet . 2012. “Who Is Eating What: Diet Assessment Using Next Generation Sequencing.” Molecular Ecology 21, no. 8: 1931–1950. 10.1111/j.1365-294X.2011.05403.x. 22171763

[ece374614-bib-0040] Powell, E. C. , C. J. Painting , A. J. R. Hickey , G. B. O. Machado , and G. I. Holwell . 2021. “Diet, Predators, and Defensive Behaviors of New Zealand Harvestmen (Opiliones: Neopilionidae).” Journal of Arachnology 49, no. 1: 122–140. 10.1636/joa-s-20-002.

[ece374614-bib-0041] Read, V. M. S. J. , and R. N. Hughes . 1987. “Feeding Behaviour and Prey Choice in *Macroperipatus torquatus* (Onychophora).” Proceedings of the Royal Society of London. Series B, Biological Sciences 230: 483–506. 10.1098/rspb.1987.0030.

[ece374614-bib-0042] Roze, J. A. 1982. “New World Coral Snakes (Elapidae): A Taxonomic and Biological Summary.” Memorias del Instituto Butantan 46: 305–338.

[ece374614-bib-0043] Smokoska, M. , and V. J. Acosta‐Chaves . 2017. “ *Trimetopon slevini* Dunn, 1940. Predation on a Velvet Worm.” Mesoamerican Herpetology 4, no. 2: 438–441.

[ece374614-bib-0044] Thompson, R. M. , U. Brose , J. A. Dunne , et al. 2012. “Food Webs: Reconciling the Structure and Function of Biodiversity.” Trends in Ecology & Evolution 27, no. 12: 689–697. 10.1016/j.tree.2012.08.005. 22959162

[ece374614-bib-0045] Toft, S. , C. Pavón‐Peláez , M. Martinez‐Villar , et al. 2021. “Contrasting Patterns of Food and Macronutrient Limitation in the Field Among Co‐Existing Omnivorous Carnivores.” Ecological Entomology 46, no. 4: 898–909. 10.1111/een.13026.

[ece374614-bib-0046] Villarreal Manzanilla, O. , J. Manzanilla , and F. Steines . 2008. “Dos casos de anurofagia por *Santinezia curvipes* (Roewer, 1916) (Opiliones: Cranaidae).” Boletín de la Sociedad Entomológica Aragonesa 1, no. 42: 317–319.

[ece374614-bib-0047] Walker, T. J. , and S. Masaki . 1989. “Natural History.” In Cricket Behavior and Neurobiology, edited by F. Huber , T. E. Moore , and W. Loher , 1–42. Cornell University Press.

[ece374614-bib-0048] Wise, D. H. 1993. Spiders in Ecological Webs. Cambridge University Press.

